# The regression models for lifelong learning competencies for teacher trainers

**DOI:** 10.1016/j.heliyon.2023.e13749

**Published:** 2023-02-16

**Authors:** Win Phyu Thwe, Anikó Kálmán

**Affiliations:** a Doctoral School of Education, University of Szeged, Szeged, Hungary; b Department of Technical Education, Budapest University of Technology and Economics (BME), Budapest, Hungary

**Keywords:** Regression model, Lifelong learning, Lifelong learning competencies, Teacher trainer

## Abstract

Contemporary youth require novel talents and fluencies due to the profound changes that are taking place in society. From school education to professional development and lifelong learning, everyone needs to have twenty-first-century skills to cope with these changes and the new normal. Lifelong learning should be the guiding idea for the future revitalization of the teaching profession. The development of lifelong learning competencies will enable teachers to help their students become lifelong learners. Teacher education is undoubtedly the most important component for teachers who seek to gain lifelong learning competencies. The study of teacher education is essential for investigating the factors that affect lifelong learning competencies among teacher trainers. The main aim of this study is investigating whether perception of lifelong learning and learning strategies could explain teacher trainers’ lifelong learning competencies and to examine whether their professional and personal factors could influence this. For this study, a correlation research design was chosen. The research sample was made up of 232 teacher trainers from various education degree colleges in Myanmar using random sampling method. Multiple linear regression analysis was performed to formulate the regression models for lifelong learning competencies of teacher trainers, and analysis of variance was also utilized to create comparison between the outcome models. The results indicated that the including region, teaching experience, perception of lifelong learning, and learning strategies may be the best regression model for predicting lifelong learning competencies in teacher trainers. This research may also be useful for establishing practical policy to implement the lifelong learning competencies within the formal and non-formal education sectors.

## 1 Introduction

Technology, education, health, economy, agriculture, and science are all changing rapidly in the contemporary world. It is impossible to know what will happen in the future. Some current theories are likely to be partially wrong. To describe changes in assumptions, Ref. [1] introduced the concept of the paradigm shift. He used this term to refer to science, but his idea is now used in reference to many academic fields, including science, medicine, technology, education, sociology, and philosophy.

Today's youth require talents and new fluencies due to the profound changes that have taken place in our society. Education faces these challenges as well. Because the skills required to have a place in today's workforce change so quickly, the ability to work and live together must constantly be rebuilt. No education system can keep up with this necessity. However, similar skills are required across formal education to career advancement and lifelong learning, such as collaboration, critical thinking, communication, creativity, and problem-solving, which can be expected as twenty-first-century skills to live with the changes and the new normal. The guiding goal for the future revitalization of the teaching profession should be lifelong learning.

The development of a lifelong learning policy includes improved and focused in-service education for teachers as a key component [2]. To deal with the issues of the globalized era, it is essential that teachers be lifelong learners. A lifelong learner embraces learning and possesses the competencies necessary for their lifetime. Lifelong learners are also flexible and happy in adapting themselves to changes caused both by themselves and by their social environment [3]. Restructuring the education system is essential to constructing a society of lifelong learners [4]. It provides teachers with newfound abilities, motivates them, and inspires them to continue learning throughout their lives. As a result of this, it assists teachers to become into passionate learning enthusiasts who are receptive to new concepts. School environments can be made more effective by teachers who are well qualified with lifelong learning skills. A teacher's lifelong learning skills are closely associated with the quality of the educational system and with the quality of the teacher. Teachers have important role to play in educating lifelong learners. Developing learning competencies will help teachers support their students in becoming lifelong learners. The teacher education pillar is undoubtedly the most important component for teachers in gaining lifelong learning competencies. Helping prospective teachers become lifelong learners will enable them to be professionals and develop lifelong learning competencies [5]. It is imperative that teacher trainers develop these competencies first if they are to nurture future teachers. Therefore, research in teacher education is essential to investigate the factors that are affecting lifelong learning competencies of teacher trainers [2].

### 1.1 Significance of the study

Several scholars have identified the factors that can affect lifelong learning abilities, such as the work of [6] on tendencies for lifelong learning among university students. Reference [7] investigated perceptions of lifelong learning and suggested that education can be organized to ensure quality and lifelong learning. Reference [8] found four aspects that can contribute to the development of lifelong learning skills: curricular structure, the institution's resources, learning and instruction, and the assessment system. Reference [9] pointed out the importance of learning strategies in lifelong learning. Reference [10] described the importance of external barriers for explaining inequalities in students' lifelong learning participation. Reference [11] discovered the forecast of personal learning environment management in fostering lifelong learning. Reference [12] investigated interpersonal communication in the learning and teaching environment as a key indicator for current and future engagement in lifelong learning. Reference [13] studied how individual and organizational factors influence the lifelong learning competencies of elementary teachers. Reference [14] studied the role of personality traits and metacognitions in the acquisition of lifelong learning competency [15]. predicted certain demographic and professional variables in relation to occupational burnout to measure teachers' lifelong learning tendencies. Reference [16] discovered the challenges in open universities in terms of improving the lifelong learning. Reference [17] investigated the relationships between teachers' teaching beliefs, lifelong learning affinities, and change tendencies. Reference [18] evaluated chemical engineering students' lifelong learning competencies during online learning in relation to the COVID-19 pandemic. Reference [19] examined the lifelong learning tendencies of English teachers, their professional competencies, and their self-efficacy in integrating technology [20]. examined the impact of the pandemic on the lifelong learning skills of university students. Reference [21] predicted assessments that fostered the improvement of metacognition abilities and encouraged lifelong learning. Additionally, Ref. [22] indicated that people should participate in lifelong learning activities regardless of their formal education, and their awareness of their own learning should be improved.

In these studies, researchers considered the effect of background factors such as age, educational status, gender, years of work experience, the overall number of learners being taught, the type of school, and the socioeconomic status of the participants on lifelong learning competencies. However, a discrepancy has arisen between researchers’ findings in relation to the impact of demographic factors on lifelong learning competencies. For example, Ref. [18] found that gender does not have an effect on lifelong learning competencies, while [19] found that lifelong learning competencies did differ by gender. Further investigation of this point is necessary.

Previous studies have also had a gap in their participant populations. Most have examined students, primary school teachers, undergraduates, postgraduates, student teachers, teacher educators, administrators, and academic staff, but not much studies were seen in teacher education that examined the factors that contribute to teacher trainers' lifelong learning competencies, with the single exception of [7]. It is also necessary, to check the variables in the literature, as perceptions about lifelong learning and learning strategies may also influence lifelong learning capabilities. It has not been determined whether lifelong learning competencies are affected by the perceptions of lifelong learning and learning strategies together. With reference to these research gaps, we intended in this study to investigate whether perceptions of lifelong learning and learning strategies could explain teacher trainers’ lifelong learning competencies, as well as whether their professional and personal factors could influence these.

## 2 Conceptual framework

### 2.1 Lifelong learning

The broad idea of lifelong learning emerged in the 1970s and had become a topic of widespread interest in the twenty-first century. Lifelong learning is covered by a range of definitions and concepts. In 1996, UNESCO commissioned the Delor's report on lifelong learning [23], the first policy development in the field. The concept of lifelong learning is a key to the twenty-first century, according to this report. The Organisation for Economic Cooperation and Development (OECD) is working on the first two pillars of Delor's report, which emphasizes lifelong learning. In its simplest form, lifelong learning refers to all activities that are developed to upgrade the knowledge and skills of those who participate in them from infancy until death [24].

According to Ref. [25], lifelong learning is more than training or continuing education. A lifelong learning system, on this conception, should enable a variety of learning opportunities, including the exploration of conceptual understanding and the narrowing of the practical application of knowledge, through a variety of contexts, such as academic education, informal lifelong learning, and technical and industrial training. Lifelong learning is a continually stimulating process that encourages and equips people to learn all the knowledge, values, abilities, and understanding that they will require throughout their lives and that they will use in a range situation, function, and context, according to the European Lifelong Learning Initiative [26].

The components of lifelong learning and its definition vary among academics. Drawing on different perspectives on the concept of lifelong learning, scholars described the important dimensions of lifelong learning as profession, social integration and engagement, and personal growth and autonomy [27]; personal dimension, professional dimension, and political dimension [28]; or lifelong learning for teachers and higher and higher levels of education and harmonization of learning activities [29].

A precise theory of lifelong learning is still lacking, although the concept and its elements have developed significantly. A review of the literature shows that scholars attempted to apply a variety of theories to lifelong learning, including the comprehensive theory [30], the theory of transformative learning [31], and theories of societal learning [32]. A theory of lifelong learning, according to Ref. [25], must take into account the new learning structures needed to keep up with the rapid and significant changes in the nature of educational and occupational requirements. It must facilitate a range of learning opportunities, including the initial inquiry of conceptual comprehension and the condensation of the practical application of knowledge. Lifelong learning as a policy statement would struggle to achieve whatever objectives it promises due to a lack of specific ideas and sparse empirical research, according to Ref. [33]. Reference [34] posits that connectivism and generativism can better characterize and explain lifelong learning in the technological age, although no single theory of learning can adequately handle all forms of lifelong learning.

### 2.2 Lifelong learning competencies

The specific knowledge that students gain during their formal schooling process does not include lifelong learning skills [35]. Reference [36] defines lifelong learning skills as that which enables individuals to continue their own learning even after they complete formal education. These competences are used by teachers to boost their professional performance and personal growth.

Other researchers have characterized various dimensions of lifelong learning competencies. Taking into account the needs for a fulfilling personal life, a healthy standard of living, employment, active citizenship, and civic participation, the European Union Commission established eight competencies as recommended, namely, literacy, linguistic diversity, mathematical and scientific skills, digital competencies, the capacity to learn new skills, innovation, active citizenship, and expression of cultural diversity [37]. In relation to these eight competences, Ref. [38] proposed six dimensions of lifelong learning competencies: as decision-making competences, competencies of collecting information, competencies of self-management, learning how to learn competencies, digitally competence, and innovation competencies. In addition, lifelong learning competencies are among teacher competencies. The general framework of teacher competencies has been described in nine dimensions, namely competencies in the profession, in research, in curriculum, in lifelong learning, in culture, in emotion, in communication, in ICT, and in environment [36]. She explained that for self-improvement and career development, educators must possess these abilities.

### 2.3 Learning strategies

Certain standards must be held in common for the reorganization of current education systems and the construction of lifelong learning. Reference [39] described the best-known standards as follows.

• The orientation of the education system should be based on the individual, their unique personalities, and their basic needs, of which the primary needs are lifelong self-improvement, self-development, and self-realization.
• It should be possible for every individual to access any form and level of education, regardless of their social status, nationality, race, or physical condition.
• Such education must be flexible, responsive to the educational needs of the population, and focused on special interests, learning styles, and rates of potential students learning.
• Every individual must have the right to choose their own strategy for further education after graduation among the variability of educational services.
• The integration of different education types and the creation of a holistic educational segment must engage the adult population of the country as students.
• At any stage in their lives, individuals should be able to use information technology to enhance their education.

According to these standards, it can be seen that learning strategies have played an important role in determining lifelong learning and lifelong learning competencies. A variety of learning strategies exist, such as cooperative learning strategies, collaborative learning strategies, repetitive learning strategies, and self-regulated learning strategies. Learning strategies are described in our study as overall learning strategies. Learner practices that are meant to affect how they process learning are known as learning strategies [40]. Reference [41] indicated the importance of learning strategies. The basis for how people typically learn is formed early in life, as are the learning abilities that later develop. A person's learning style in later life is significantly influenced by their childhood strategies and practices. Many studies have focused on the relationship between learning strategies and academic achievement [42,43]. Reference [44] showed that learning strategies are important for success after university and in the workplace, and their impacts are not limited to academic achievement. Therefore, learning strategies should consider lifelong learning and the implementation of lifelong learning competencies.

### 2.4 Context of the study

There is no strategic plan to implement lifelong learning competencies for the teachers in Myanmar. There are several promising examples from Southeast Asian nations, including Myanmar, as indicated in a report [45] that outlines the essential characteristics for creating a culture of lifelong learning for all. In addition to promoting equitable and comprehensive quality education, these initiatives are intended to resolve the region's educational challenges. Various lifelong learning policies and strategies are found in collections of policy documents [45]. The lifelong learning policies and strategies developed by Myanmar are not found in that collection.

However, Ref. [45] reported that the first step in implementing lifelong learning in Myanmar has begun with alternative education. In 2016, the Department of Alternative Education was established, which draws an alternative education pathways map. Its mission is to provide particular groups with equally affordable, accredited, high-quality education that will develop their skills for the future development and long-term viability of Myanmar society. This new approach to education offers students a variety of possibilities to pursue their career ambitions and other incentives for lifelong learning. This strategy was developed in accordance with the Myanmar National Education Law, which prescribes that a main principle of education is that every citizen has the right to education and opportunities for lifelong learning shall be created. It establishes equivalency programs for the non-formal and formal education systems and makes important policy commitments such as for basic literacy programming, implementation, and the opportunities for lifelong learning with local and nongovernmental partners [46].

Reinforcing the lifelong learning of teacher trainers is essential to be able to produce quality teachers. Reference [28] showed that university educators' academic advancement is an ongoing process that is built on the idea of lifelong learning. Teacher education colleges have an important role to play here in helping teachers develop lifelong learning competencies.

## 3 Aims and research questions

This study's main purpose is to determine whether perceptions of lifelong learning and learning strategies can be used to explain teacher trainers' lifelong learning competencies and establish whether personal and professional factors also have an effect on them. The following specific research questions guide this study.

1. Do perceptions of lifelong learning, learning strategies, and background factors affect the lifelong learning competencies of teacher trainers?
2. How far do personal factors, perceptions of lifelong learning, and learning strategies predict lifelong learning competencies?
3. How far do professional factors, perceptions on lifelong learning, and learning strategies predict lifelong learning competencies?
4. Which prediction model is the most appropriate for lifelong learning competencies?

## 4 Method

### 4.1 Research design and procedure

This study involves a correlation research method. Because the goal of this study is to determine variables that will predict a result or set of criteria [47], a correlation research method was chosen. Based on the research gap noted above, we conducted this research with teacher trainers. Before distributing questionnaires, we identified the appropriate research tools and question items, adapted and translated them, and then established their reliability and validity.

The Institutional Review Board of the Doctoral School of Education, University of Szeged approved the study in accordance with institutional ethical guidelines. In order to distribute the questionnaires, we attempted to obtain permission from the principals of Education Degree College. All participants were informed that their participation was voluntary, and their answers would remain confidential. They were made aware of the objectives of the study, the types of instruments being used, and the proper way to complete each instrument. Further, they also informed that their participation would not be used for anything outside the research activity.

### 4.2 Participants

The population of this research was teacher trainers working at education colleges in Myanmar. There are 25 education colleges in Myanmar: 13 in upper Myanmar and 12 in lower Myanmar. We applied the most basic of the probability sampling techniques, simple random sampling. There are 1058 teacher trainers at these 25 education colleges, to an administrative officer at the Ministry of Education, Myanmar. The research sample was composed of 232 teacher trainers at different education degree colleges in both regions. This sample size is acceptable at 95% confidence level, where sampling error is permitted between 5% and 8% in social science. Table 1 shows the demographic information. It should be noted that the number of female teacher trainers was much more than the male, reflecting the broader gender pattern in teacher education in Myanmar.

**Table 1:** Demographic factors of teacher trainers.

Participants' demographic factors			frequency	%
Personal factors	Gender	Male	25	10.76%
		Female	207	89.22%
	Age	20–30 years	57	24.57%
		31–40 years	88	37.93%
		41–50 years	32	13.79%
		Over 50 years	55	23.71%
Professional factors	Region	Lower	60	25.86%
		Upper	172	74.14%
	Education level	Bachelor	51	21.98%
		Master	167	71.98%
		Phd	14	6.03%
	Teaching Service	1–5 years	88	37.93%
		6–10 years	64	27.59%
		11–15 years	37	15.95%
		Over 15 years	43	18.53%
Total			232	100%

### 4.3 Instrument

The following three research instruments were used to investigate our research questions. The psychometric properties of the questionnaires were also evaluated.

#### 4.3.1 Lifelong Learning Questionnaire

To examine the perceptions of teacher trainers on the lifelong learning, we used the Perceptions of Lifelong Learning Questionnaire, which was developed by Ref. [7]. After adapting and translating it into the context of our study, this instrument is one dimensional and composed of 9 items (e.g. “Lifelong learning can improve personal and professional developments.”). Its reliability was high, α = 0.84 and model fitness are also acceptable (Satorra–Bentler chi-square = 86.67; df = 27; p ≤ .001; robust CFI = 0.86; robust TLI = 0.81; robust RMSEA = 0.09; SRMR = 0.07).

#### 4.3.2 Lifelong Learning Competencies Scale

The 27-items Lifelong Learning Competencies Scale (LLLCS), based on the European framework, was used to investigate the lifelong learning competencies of teacher trainers. The LLLCS has eight domains, namely, literacy competence, with three items (e.g. “aware of the main types of verbal interaction, a range of literary and non-literary texts, and the main features of different styles and registers of the Myanmar language”); multilingual competence, with three items (e.g. “appreciate of cultural diversity, an interest and curiosity about different languages and intercultural communication”); mathematical competence and competence in science, technology, and engineering, with six items; digital competence, with three items; learning to learn competence, with three items; citizenship competence with three items; entrepreneurship competence, with three items; and cultural awareness and expression competence with three items. We checked the instrument's reliability and validity, finding that it had high reliability (α = 0.89) and acceptable validity (Satorra–Bentler chi-square = 381.014; df = 296; p ≤ .001; robust CFI = 0.92; robust TLI = 0.91; robust RMSEA = 0.05; and robust SRMR = 0.06).

#### 4.3.3 Teachers’ Learning Strategies Questionnaire

To identify the learning strategies used by the participating teacher trainers, the Learning Strategies scale derived from the Teaching and Learning Strategies Questionnaire designed by Ref. [48] was used, which consists of 16 items (e.g. “Set own learning goals”). The original questionnaire includes four subscales: students' learning strategies and approach to teaching, portfolio use, and technology experience. In accordance with the context of the study, we adapted students' learning strategies into teachers’ learning strategies. The scale had high reliability (α = 0.92) and good fitness (Satorra–Bentler chi-square = 224.86; df = 104; p ≤ .001; robust CFI = 0.90; robust TLI = 0.82; robust RMSEA = 0.07; SRMR = 0.08).

These questionnaires were graded on a 4-point Likert scale ranging from 1 (strongly disagree) to 4 (strongly agree). When we collected these data from the teacher trainers, we also collected the demographic information, in an instrument with dichotomous and multiple-choice questions to obtain personal and professional information regarding gender, age, region of the education degree colleges, level of education, and teaching services as a teacher trainer.

### 4.4 Data analysis

The data were analyzed using the statistical package of R version 4.1.0 (2021-05-18). Reference [47] suggested multiple linear regression analysis was appropriate for the prediction research design. Multiple linear regression analysis (MLRA) was carried out with lifelong learning competencies as the dependent variable and personal and professional variables, including perception of lifelong learning and learning strategies, as the independent variables. First, we analyzed all of the independent variables together to address the first research question. Second, we chose the personal factors (gender and age), perception of lifelong learning, and learning strategies to address the second research question. Third, we selected professional factors (college region, education level, and teaching service), and perception of lifelong learning and learning strategies to solve the third research question. As a result of computing the MLRA three times, three alternative regression models were derived. To evaluate the multicollinearity among the predictors of these three regression models, variance inflation factor (VIF) values were also taken into consideration. Finally, analysis of variance (ANOVA) was also used to compare the outcome models and decide the best model to address the fourth research question.

## 5 Results

To predict the LLL competencies of the teacher trainers, all variables, including both personal (gender and age) and professional (education level, regions, and teaching experience) factors, perceptions of LLL, and learning strategies were treated as independent variables, while the LLL competencies are considered the dependent variable. According to multiple linear regression coefficients, it was found that perceptions of LLL (β = 0.46, p < .001) and learning strategies (β = 0.22, p < .001) were significant positive predictors, while the location of the education degree college (β = −0.051, p < .01) was a significant negative predictor. The overall model fit was R2 = 0.73, meaning that the three independent variables explained 73% of the differences in the acquisition of the acquisition of lifelong learning competencies. These three independent variables predict LLL competencies significantly well; F (224) = 89.75, p < .001. The results of the model are summarized in Eq. (1). This model indicates that a 0.46% increase (±0.029907) in the lifelong learning competencies is correlated with a 1% increase in perception of lifelong learning, a 0.22% increase (±0.032770) in the lifelong learning competencies is associated with a 1% increase in learning strategies, and a 0.05% (±0.0320057) decrease in lifelong learning competencies is correlate with a 1% increase based on the region of the college. This is our first model of lifelong learning competencies.

(1) LLLcompetencies=103+(0.46*perceptiononLLL)+(0.22*learningstrategies)–(0.05*region)±0.121781

To develop the second model, personal factors, such as gender, age, perception of lifelong learning, and learning strategies were considered as independent variables. The MLRA showed that perceptions of LL (β = 0.46, p < .001) and learning strategies (β = 0.22, p < .001) were significant positive predictors. Using these two independent variables, LLL competencies could be predicted significantly well: F (227) = 105.7, p < .001. Overall, R2 = 0.72, indicating that these two independent variables explained 72% of the lifelong learning competencies. The resulting model described in Eq. (2) means that lifelong learning competencies increase by 0.46% (±0.029916) for every 1% increase in perception of lifelong learning, and they increase by 0.22% (±0.032970) for every 1% increase in learning strategies.

(2) LLLcompetencies=93+(0.46*perceptiononLLL)+(0.22*learningstrategies)±0.114392

For independent variables in the third model, professional factors (education level, region, and teaching experience), perception of lifelong learning, and learning strategies were considered. The results showed that perceptions regarding LL (β = 0.46, p < .001) and learning strategies (β = 0.23, p < .001) were significant positive predictors, whereas the region of the education degree colleges (β = −0.051, p < .01) and teaching experience (β = −0.01, p < .01) were significant negative predictors. Taking these four predictors into account, 73% of the variation in lifelong learning competency development (R2 = 0.73) can be explained. The predictors have a significant influence on LLL competencies: F (226) = 126.4, p < .001. The outcome model as shown in Eq. (3) indicates that 1% more perception of lifelong learning is corelated with a 0.46% (±0.029627) increase in lifelong learning competencies, a 1% increase in learning strategies is corelated with a 0.23% (±0.032210) increase in lifelong learning competencies, and a 1% increase based the region of the college is correlated with a decrease in lifelong learning competencies by 0.05% (±0.019950), while a 1% increases in teaching experience is correlated with a decrease in lifelong learning competencies by 0.01% (±0.007236).

(3) LLLcompetencies=108+(0.46*perceptiononLLL)+(0.22*learningstrategies)–(0.05*region)–(0.01*experience)±0.102910

To check for multicollinearity between the predictors of each model, the VIF was computed. All variables in each model with VIFs lower than 2 showed no multicollinearity, according to Ref. [49]. ANOVA was used to compare the three models. When comparing the first and second models, the resultant p-value (p < .01) is sufficiently low (usually smaller than 0.05). Accordingly, the more complex model (first model with region, perception of lifelong learning, and learning strategies) outperforms the simpler model (the second model). Comparing second and third models showed a significant difference (p < .001) between them, so a more complex model (the third model with region, and teaching experience, perception of lifelong learning, and learning strategies) was preferred. Furthermore, no significant difference was found between first and third models. While R2 values were the same for both the first and third models, the standard error of regression of the third model (ε = 0.102910) was the least for it among all three models.

## 6 Discussion

The literature clearly shows a wide range the impact of personal and professional factors, perception of lifelong learning, and learning strategies in relation to LLL competencies. In general, all of our regression models indicated that perceptions regarding lifelong learning and learning strategies are significant predictors of lifelong learning competencies. The regression models of this study were aligned with those of previous studies that examined attitudes and LLL competencies [50], knowledge sharing and LLL competencies, learning readiness [13], learning activities [51], learning strategies [9,24,52]. On the other hand [18] found that perception does not affect lifelong learning competencies, although interest and receptivity to learning matter to LLL competencies. Knowing and understanding one's preferred learning strategies is important in all contexts. To achieve specific work or career goals, learners must be aware of their own competencies, abilities, and qualifications [37,53].

All three of our regression models indicated that lifelong learning competencies are not related to the personal factors such as gender and age. Several studies have shown that lifelong learning competencies do not differ by gender [18,51,54,55] and age [19,56] while these studies suggest differences between lifelong learning abilities in terms of gender [4,13,19].

When professional factors are taken into account, lifelong learning competencies also depend on the region, according to our first and third models. In addition to region, the third model of this study includes teaching services as a factor that can predict lifelong learning competencies. These two models are in line with previous studies showing that lifelong learning competencies differ by region [15] and teaching service [15,57,58]. It is worth noting that our models contrast with these studies, which show that the following professional factors are not related to lifelong learning competencies: region of the college [19,51,56] and teaching service [19,51,54]. Among the professional factors we examined, level of education is not very important for learning competencies in our models. This finding contradicts a previous study that reported a significant difference between lifelong learning competencies by education level [56]. On the other hand, other past studies did find that education level does not affect lifelong learning competencies [18,19,51].

After assessing these three models, we compared using computing their VIFs and ANOVA analysis to determine the best model. The values of VIFs showed that there was no multicollinearity among the variables of any model. The ANOVA could not determine the best model among the three. When the standard error of regression was also taken into account, the third model was shown to have the lowest error. These findings show the third model with region, teaching experience, perception of lifelong learning, and learning strategies may be the best regression model for predicting LLL competencies in teacher trainers. In other words, lifelong learning competencies are influenced by the region, teaching experience, perception on LLL, and learning strategies.

Using the third model, we discuss investigate possible possibilities that have been influenced by the context of the study, such that the relationship between region, year of teaching experience, and LLL competencies of teacher trainers are reciprocal factors. In certain regions, educational degree colleges have only been operating in the last few years and have inadequate facilities and resources, which may impact those who work there. In the OECD reports as well, it is noted that external assistance, such as that provided by higher education institutions, educational centers, and regional or specialized support teams, plays an important role. It is particularly important in workplaces where geographic and occupational isolationism pose a threat [2]. Additional teaching experience relates to higher responsibilities and the lower motivation to learn. Reference [15] suggested that regular in-service training should be provided to teachers to eliminate a chasm in LLL tendencies based on regional differences and teaching experience.

This research may be useful for establishing a practical policy to implement the lifelong learning competencies within the formal and non-formal education sectors. According to the World Bank, establishing effective lifelong learning programs require significant improvements to both administration and funding in both education and training. In many OECD countries, governments are already working to develop adaptive policy and regulatory structures that incorporate a broader spectrum of institutional actors, which had previously been based only on public funding and public education [59]. The necessity of creating national strategies for lifelong learning was highlighted by the European Commission when it evaluated the results of the education reforms in 2008. It is also important to take local issues into consideration when establishing the lifelong learning in Myanmar. National lifelong learning strategies should involve step-by-step guidelines that focus on teacher education, providing awareness, and formal and non-formal training, as well as facilitating opportunities for informal learning.

Our regression models indicate that it would be beneficial to provide teacher trainers with training activities to raise their awareness of lifelong learning. Enhancing teacher trainers' perception of lifelong learning will enable them to upgrade key lifelong learning competencies. The lifelong learning competencies of teacher trainers should also be promoted by encouraging them to adopt their preferred learning strategies. When appropriate learning strategies are determined, trainings that support the implementation of lifelong learning competencies will be more effective. Our best regression model suggests that all of these strategies should be implemented with care, such that experienced service teacher trainers from all regions be incorporated. Higher lifelong learning competencies in teacher trainers provide superior opportunities for student teachers at education degree colleges to gain those competencies. These student teachers will certainly become basic education teachers, and they will need to produce young students with the updated competencies needed to continue to stay on top of the world's changing trends.

## 7 Limitations and suggestions

A number of drawbacks in this study are important to mention. First of all, our sample was different from that of past comparable studies, which have primarily focused on school teachers, university teachers, college students, and prospective teachers, whereas ours is composed of teacher trainers working in teacher education. This may have led to some variations in interpretation between samples. Second, the eight separate domains of the LLL competencies are not measured in detail in relation to personal and professional factors, perceptions of LL or learning strategies.

The conflicting findings of this study indicate that further investigation is needed to consider the lifelong learning competencies of teacher trainers in the contexts of different countries. In future studies, it will be possible to examine how perceptions regarding LLL and/or learning strategies affect the different domains of LLL competencies. The relationship between the literacy competence that belongs to LLL competencies and perception of LLL, for example, is also worth considering. It is likely, however, that the findings of this present study will also be helpful to close the disparity in lifelong learning research in teacher education. An example of possible variety of results could be produced, depending on the context, though a follow-up interview, providing an opportunity to identify further factors that might contribute to or hinder LLL competencies.

## 8 Conclusion

This study was conducted to predict the impact of personal and professional factors, perception of lifelong learning, and learning strategies on lifelong learning competencies of teacher trainers. The results show that personal and professional factors, as well as gender, age, and education level, play little role in determining lifelong learning competencies. Lifelong learning competencies depend on the region where the given individual is working, their teaching experience, how they perceive the lifelong learning, and how they use learning strategies for their teacher competencies. Our findings show some differences from previous findings, with potential contextual justifications for our findings. In addition, our findings may prove useful both on a national and practical level. Our study had acknowledged limitations, which lead to suggestions for further research.

## Author contribution statement

Win Phyu Thwe: Conceived and designed the experiments; Performed the experiments; Analyzed and interpreted the data; Contributed reagents, materials, analysis tools or data; Wrote the paper.

Anikó Kálmán: Analyzed and interpreted the data; Wrote the paper.

## Funding statement

This work was supported by Uniersity of Szeged/ Szegedi Tudományegyetem [5919].

## Data availability statement

The data that has been used is confidential.

## Declaration of interest's statement

The authors declare no competing interests.

## References

[1] Kuhn T.S. (1997).

[2] Coolahan J. (2002). http://ideas.repec.org/p/oec/eduaab/2-en.html.

[3] Demirel M. (2009). Implications of lifelong learning on educational institutions. Cypriot J. Educ. Sci..

[4] Pilli O., Sönmezler A., Göktan N. (2017). Pre-service teachers' tendencies and perceptions towards lifelong learning. Eur. J. Soc. Sci. Educ. Res..

[5] Selvi K. (2011). Teachers' lifelong learning competencies. Int. J. Curric. Instr. Stud..

[6] Bath D.M., Smith C.D. (2009). The relationship between epistemological beliefs and the propensity for lifelong learning. Stud. Cont. Educ..

[7] Buza L., Buza H., Tabaku E. (2010). Probl. Educ. 21st Century.

[8] Latif L.A., Fadzil M., Goolamally N. (2012). The 40th Anniversary of KNOU Future of ODL for ‘Knowledge Network Society’ Korea.

[9] Müller R., Beiten S. (2013). Changing learning environments at university? Comparing the learning strategies of non-traditional European students engaged in lifelong learning. J. Educ. Sci. Psychol..

[10] Lavrijsen J., Nicaise I. (2017). Systemic obstacles to lifelong learning: the influence of the educational system design on learning attitudes. Stud. Cont. Educ..

[11] Yen C.J., Tu C.H., Sujo-Montes L.E., Harati H., Rodas C.R. (2019). Using personal learning environment (PLE) management to support digital lifelong learning. Int. J. Online Pedagog. Course Des..

[12] Deveci T. (2019). Interpersonal communication predispositions for lifelong learning: the case of first year students. J. Educ. Fut..

[13] Shin Y.-S., Jun J. (2019). The hierarchical effects of individual and organizational variables on elementary school teachers' lifelong learning competence. Int. Electron. J. Environ. Educ..

[14] Grokholskyi V.L., Kaida N.I., Albul S.V., Ryzhkov E.V., Trehub S.Y. (2020). Cognitive and metacognitive aspects of the development of lifelong learning competencies in law students. Int. J. Cogn. Res. Sci. Eng. Educ..

[15] Yildiz-Durak H., Seferoglu S.S., Sen N. (2020). Some personal and professional variables as identifiers of teachers' lifelong learning tendencies and professional burnout. Cypriot J. Educ. Sci..

[16] Zuhairi A., Hsueh A.C.T., Chiang I.-C.N. (2020). Empowering lifelong learning through open universities in Taiwan and Indonesia. Asian Assoc. Open Univ. J..

[17] Karataş K., entürk C., Teke A. (2021). The mediating role of self-directed learning readiness in the relationship between teaching-learning conceptions and lifelong learning tendencies. Aust. J. Teach. Educ..

[18] Yap J.S., Tan J. (2022). Lifelong learning competencies among chemical engineering students at Monash University Malaysia during the COVID-19 pandemic. Educ. Chem. Eng..

[19] Sen N., Durak H.Y. (2022). Examining the relationships between English teachers' lifelong learning tendencies with professional competencies and technology integrating self-efficacy. Educ. Inf. Technol..

[20] Deveci T. (2022). UAE-based first-year university students' perception of lifelong learning skills affected by COVID-19. Tuning J. High. Educ..

[21] Matsumoto-Royo K., Ramírez-Montoya M.S., Glasserman-Morales L.D. (2022). Lifelong learning and metacognition in the assessment of pre-service teachers in practice-based teacher education. Front. Educ..

[22] Arslan A. (2022). The effect of university students' achievement orientations on lifelong learning tendencies: a structural equation model study TT – Üniversite Öğrencilerinin Başarı yönelimlerinin yaşam Boyu Öğrenme Eğilimlerine Etkisi: yapısal Eşitlik modeli Çalışması. Çukurova Univ. Fac. Educ. J..

[23] Delors J., Chung F., Gorham W., Manley M. (1998). https://books.google.com.ph/books?hl=en&lr=&id=tGXxgar1fCQC&oi=fnd&pg=PA1&dq=Learning:+The+Treasure+Within&ots=sVl9uS6LaV&sig=MPQ9wuSkf2p9sr06CrYbsuw0664&redir_esc=y#v=onepage&q=Learning%3A%20The%20Treasure%20Within&f=false.

[24] Gonczi A. (2007). Graduate Attributes, Learning and Employability.

[25] Fischer G. (2000). Lifelong learning—more than training. J. Interact. Learn. Res..

[26] Watson L. (2003). https://www.semanticscholar.org/paper/Lifelong-learning-in-Australia-Watson/30be62bbe2448a4f9df723b70c2e2ab0f96cc854.

[27] Shrestha M., Wilson S., Singh M. (2008). Knowledge networking: a dilemma in building social capital through nonformal education. Adult Educ. Q..

[28] Smith C.R. (2015).

[29] Kálmán A. (2016).

[30] Bagnall R.G. (2017). A critique of Peter Jarvis's conceptualisation of the lifelong learner in the contemporary cultural context. Int. J. Lifelong Educ..

[31] Eschenbacher S., Fleming T. (2020). Transformative dimensions of lifelong learning: Mezirow, Rorty and COVID-19. Int. Rev. Educ..

[32] Osborne M., Borkowska K. (2017). A European lens upon adult and lifelong learning in Asia. Asia Pac. Educ. Rev..

[33] Edwards R., Ranson S., Strain M. (2002). Reflexivity: towards a theory of lifelong learning. Int. J. Lifelong Educ..

[34] Steffens K. (2015). Competences, learning theories and moocs: recent developments in lifelong learning. Eur. J. Educ..

[35] Dong W. (2004). Improving students' lifelong learning skills in circuit analysis. China Pap..

[36] Selvi K. (2010). Teachers' competencies. Cult. Int. J. Philos. Cult. Axiol..

[37] European Council (2006). Recommendation of the European Parliament and the Council on key competencies for lifelong learning. Off. J. Eur. Union.

[38] Uzunboylu H., Hürsen Ç. (2011). Lifelong learning competence scale (Lllcs): the study of validity and reliability. J. Educ..

[39] Burlakova I., Budnik A., Burlakova E.S. (2019). V International Forum on Teacher Education.

[40] Mayer R.E. (1988).

[41] Endres T., Leber J., Büttger C., Rovers S., Renkl A. (2021). Improving lifelong learning by fostering students' learning strategies at university. Psychol. Learn. Teach..

[42] Reyes B., Jimenez-Hernandez D., Martinez-Gregorio S., De los Santos S., Galiana L., Tomas J.M. (2022). Prediction of academic achievement in Dominican students: mediational role of learning strategies and study habits and attitudes toward study. Psychol. Sch..

[43] Vermunt J.D., Donche V. (2017). A learning patterns perspective on student learning in higher education: state of the art and moving forward. Educ. Psychol. Rev..

[44] Vilppu H., Mankki V., Lahteenmaki M., Mikkila-Erdmann M., Warinowski A. (2022). Debunking the myth of high achievers in Finnish primary teacher education: first-year preservice teachers' learning strategies and study success. Eur. J. Teach. Educ..

[45] Yorozu R. (2017). https://unesdoc.unesco.org/ark:/48223/pf0000253603.

[46] Myanmar Education Law (2014). https://www.ilo.org/dyn/natlex/natlex4.detail?p_lang=en&p_isn=100493&p_count=3&p_classification=09.

[47] Creswell J.W. (2012). https://drive.google.com/file/d/1d5ZzlgJuCrwAyLpdBeK5dhKMZTpE2HNb/view.

[48] Abrami P.C., Wade C.A., Pillay V., Aslan O., Bures E.M., Bentley C. (2005). https://files.eric.ed.gov/fulltext/EJ1073852.pdf.

[49] James G., DanielaWitten, Hastie T., Tibshirani R. (2013).

[50] Tenekeci F., Uzunboylu H. (2020). Determining the relationship between the attitudes of private teaching institution teachers towards lifelong learning and their competence. Int. J. Learn. Teach..

[51] Aykaç M., Köğce D., Aslandağ B. (2020). The investigation of mathematics teachers' perceptions of lifelong learning competencies. Educ. Policy Anal. Strateg. Res..

[52] Kavaliauskiene G., Kaminskiene L. (2009). A complementary approach to lifelong learning strategies. Ibérica Rev. la Asoc. Eur. Lenguas para Fines Específicos.

[53] Hozjan D. (2009). Key competences for the development of lifelong learning in the European Union. Eur. J. Vocat. Train..

[54] Bülbül A. (2020). Examination of lifelong learning trends of physical education and sports teachers on different variables. J. Educ. Learn..

[55] Sahin M., Akbasli S., Yelken T.Y.T.Y. (2010). Key competences for lifelong learning: the case of prospective teachers. Educ. Res. Rev..

[56] Ayanoglu C., Guler N. (2021).

[57] Bozat P., Bozat N., Hursen C. (2014). The evaluation of competence perceptions of primary school teachers for the lifelong learning approach. Procedia – Soc. Behav. Sci..

[58] Kuzu S., Demir S., Canpolat M. (2015). Evaluation of life-long learning tendencies of pre-service teachers in terms of some variables. Eğitimde Kuram ve Uygul. Artic./Makaleler J. Theory Pract. Educ..

[59] Wheeler David (2003).

