# Corrigendum to “The regression models for lifelong learning competencies for teacher trainers”

**DOI:** 10.1016/j.heliyon.2023.e14268

**Published:** 2023-03-09

**Authors:** Win Phyu Thwe, Anikó Kálmán

**Affiliations:** a Doctoral School of Education, University of Szeged, Szeged, Hungary; b Budapest University of Technology and Economics (BME), Budapest, Hungary

In the original published version of this article, the authors did not mention the acknowledgements in the main manuscript. We have now added the acknowledgements. The authors apologize for the errors. Both the HTML and PDF versions of the article have been updated to correct the errors.

